# A genome-wide association study of methamphetamine use among people with HIV

**DOI:** 10.1186/s12920-025-02105-8

**Published:** 2025-03-11

**Authors:** A. Venkataraman, T. Jia, S. A. Ruderman, C. B. Haas, R. M. Nance, L. S. Mixson, K. H. Mayer, M S Saag, G. Chander, R. D. Moore, J. Jacobson, S. Napravnik, K. Christopolous, W. J. Lee, B. M. Whitney, I. Peter, H. M. Crane, J. A. C. Delaney, S. Lindström

**Affiliations:** 1 University of Washington, Seattle, WA USA; 2 Harvard University, Cambridge, MA USA; 3 University of Alabama, Birmingham, AL USA; 4 Johns Hopkins University, Baltimore, MD USA; 5 Case Western Reserve University, Cleveland, OH USA; 6 University of North Carolina, Chapel Hill, NC USA; 7 University of California, San Francisco, CA USA; 8 Icahn School of Medicine at Mount Sinai, New York, NY USA; 9 University of Manitoba, Winnipeg, MB Canada; 10 Public Health Sciences Division, Fred Hutchinson Cancer Center, Seattle, WA USA

**Keywords:** Methamphetamine, Substance use, GWAS, HIV, Genetic epidemiology, Stimulant use

## Abstract

**Background:**

Amphetamine-like stimulants are the most used psychostimulants in the world; methamphetamine use is the most prevalent in people with HIV. Prolonged methamphetamine use can cause lasting damage to the heart, gut, and brain, as well as auditory hallucinations and paranoid thinking. However, relatively little is known about methamphetamine use and its genetic contributors.

**Methods:**

Using genetic information from the Centers for AIDS Research Network of Integrated Clinical Systems (CNICS) cohort, we conducted a multi-ancestry genome-wide association study (GWAS) of methamphetamine use among people with HIV (*n* = 1,196 reported ever use, *n* = 4,750 reported never use).

**Results:**

No single nucleotide polymorphism was statistically associated with methamphetamine use at the genome-wide level (*p* < 5 * 10^–8^) in our study. Further, we did not replicate previously suggested genetic variants from other studies (all *p* > 0.05 in our analysis).

**Discussion:**

Our study suggests that there is no single strong genetic contributor to lifetime use of methamphetamine in people with HIV enrolled in CNICS. Larger studies with more refined outcome assessment are warranted to further understand the contribution of genetics to methamphetamine use and use disorder. Investigation into social and environmental contributors to methamphetamine use are similarly necessary.

## Introduction

Amphetamine-like stimulants are the most used psychostimulants in the world. Among them, methamphetamine (meth) use is the most prevalent, and in 2022, 2.7 million people in the United States reported using meth in the last year, 176,000 of whom initiated use in that time [1]. Moreover, meth use is relatively prevalent among people with HIV (PWH), with some meta-analyses reporting a prevalence ratio for PWH as high as 1.86 compared to people without HIV [2]. While 0.9% of people aged 12 or older reported ever using meth in 2021 [3], a publication from the Centers for Disease Control and Prevention reported that 11.7% of adults with diagnosed HIV used meth from 2015–2018 [4]. However, prolonged meth use can cause lasting damage to the heart, gut, and brain, as well as auditory hallucinations and paranoid thinking [5, 6], further, people who use meth are likely to experience stigma and social alienation from their communities, due to a comparative lack of understanding about meth use and its treatment compared to other drugs and the overlap with HIV status, among other factors [7]. Disordered meth use can also be a significant financial burden on people and their social networks.

Relatively little is known about meth use and/or dependence compared to other drugs (e.g., heroin, alcohol), and only a few studies have investigated the genetic contributors of each. Moreover, no twin or family studies have been carried out examining meth use or meth use disorder heritability, though heritability studies of overall stimulant use disorder excluding cocaine have reported estimates of 0.40–0.42 [8, 9]. To date, four genome-wide association studies (GWASs) of meth use disorder have been conducted, all of which were performed in populations of Han Chinese or Japanese ancestry; however, there is evidence of ethnic divergence of gene variants for meth use disorder [10–14]. Further, these studies were carried out among overlapping populations, and total*n*ranged from 580 [14] to 4,608 [13].

Genome-wide data in combination with clinical data of the Centers for AIDS Research Network of Integrated Clinical Systems (CNICS) cohort allow for investigations into the genetic contribution to additional outcomes that are relevant to people with HIV on a national scale. We utilize the extensive CNICS infrastructure for collection of patient-reported outcomes (PROs) and ongoing collection of the CNICS clinical assessment of PROs which has resulted in nearly 70,000 assessments of adherence and substance use among ~ 16,000 PWH to date. Given the high prevalence of methamphetamine use among people with HIV and the extensive substance use and genetic data collected by CNICS, our cohort is uniquely suited to studies investigating genetic contributors to methamphetamine use. In this study, we report the first GWAS for methamphetamine use among 5,946 PWH in the CNICS cohort.

## Methods

### Study population and phenotype data

We conducted this study among PWH in the Centers for AIDS Research Network of Integrated Clinical Systems (CNICS) cohort for whom we had genotype data [15]. CNICS is a well-characterized longitudinal observational cohort of over 48,000 PWH who enrolled in care at 8 geographically distinct HIV clinics in the US from 1995 to the present (http://www.uab.edu/cnics/). The CNICS data repository integrates longitudinal clinical data from outpatient and inpatient encounters, including laboratory data, medications, diagnoses, vital status, and health care use history. CNICS participants complete a clinical assessment of patient reported measures and outcomes (PROs) at routine clinic visits every ~ 4–6 months and have completed > 103,000 clinical assessments to date. The WHO ASSIST tool, previously validated for reliability and feasibility and employed in previous CNICS studies, was used to assess lifetime non-prescription drug use (i.e. ‘In your lifetime, have you ever used…’) [16–18]. CNICS participants are also screened for alcohol use, depression/anxiety, and other domains [19]. Further, adult participants who provided informed consent were genotyped as part of an ongoing genetics project. CNICS participants were included in this study if their genetic data was available at the time of analysis and if they had completed one or more clinical assessments. IRBs at each site approved the study protocol, and all participants provided informed consent to be included in the study. Of all CNICS participants, lifetime methamphetamine (crystal meth, speed, or Tina) use data was available for 5,946 PWH. Of them, 1,196 reported lifetime use at their most recent timepoint. Drop out rate for the whole cohort is estimated at 15% per year. Among individuals who have never used meth, it is estimated at 13%, compared to 15% among former meth users.

### Genotyping and imputation

Genetic data is based on reference genome GRCh38. The extensive genotyping and quality control pipeline has been described in [16]. Briefly, genotyping was performed using the Illumina Multi-Ethnic Global Array (MEGA) and Expanded version (MEGAEx), and Infinium Multi-Ethnic Global-8 Kit (MEG). In total, 3589 samples were genotyped in MEGA; 4694 in MEGAEx; and 3017 in MEG. We performed quality control within arrays by restricting to chromosome 1–22 and removing variants and samples with a missing genotype rate greater than 5%. We removed variants with extreme departure from Hardy–Weinberg equilibrium (*p* < 1*10^–30^). We used the 1,000 Genomes Project (1KGP) data to assign each genotyped individual to continental ancestry groups [20, 21], including African (*n* = 5,051), Admixed American (*n* = 1,741), and European (*n*= 3,240) by identifying SNPs included in both our pruned dataset and in 1KGP, ignoring INDELs. Sex checks and relatedness within array and ancestry were assessed using the’check-sex’ function in PLINK v1.9 to compute X chromosome inbreeding coefficients (parameter F) in ancestry subsets. We restricted to chromosome X, removing the pseudo-autosomal region, set a genotype missing rate of > 5%, MAF < 0.05, and LD pruning (independent-pairwise 10,000 kb). We chose an F minimum of 0.5 for female cutoff, and 0.8 for males. We then merged the remaining samples across the genotyping arrays within ancestry, restricting to common SNPs using Genotype-Harmonizer [22]. To address bias by array type, we used PLINK to generate principal components (PCs) using the same pruning steps described above and tested all SNPs for associations with platform as the outcome, adjusting for 10 PCs. Significantly associated SNPs (*p* < 5*10^–8^) were removed before imputation. All data were imputed using the multi-ancestry Trans-Omics for Precision Medicine (TOPMed) reference panel [23].

### Statistical analysis

We performed genome-wide analyses within each of the three ancestry groups (AFR, AMR, EUR) for never/ever use of meth. Assuming a SNP with an average allele frequency of 30% and an additive model, we had more than 80% power to detect an overall odds ratio of 1.31 at a *p*-value of 5 × 10^–8^. We restricted analyses to variants with imputation quality score > 0.8, MAF (Minor Allele Frequency) > 0.05, and Hardy–Weinberg Equilibrium *p* > 1*10^–10^. We conducted association analyses using the GENESIS package in R [24]. We created a null model by regressing the outcome (never/ever use of meth) on the following covariates: age at visit, the first five genetic principal components, genotyping array, and with the genetic relatedness matrix as a covariate matrix for random effects. We performed a meta-analysis across the three groups using the MR-MEGA software, which is well-powered to detect associations at loci with allelic heterogeneity and requires that variants have significant overlap between input datasets [25]. Proxy SNPs for top hits were assessed using the ‘proxy-assoc’ function in PLINK v1.9. Manhattan plots were produced in Python 3 using the qmplot package [26]. We compared our results to those previously reported in Uhl et al. [14], Chang et al. [11], Ikeda et al. [12], and Sun et al. [13].

## Results

Lifetime meth use data was available for 5,946 PWH, of which 1,196 (20.1%) reported having ever used meth during the lifetime (Table 1). An average of 5 meth assessments per person was recorded, with a median value of 3 (IQR 1–7). Dropout rate among all individuals was 15%: 13% among individuals who reported never using meth, 15% among individuals who reported ever using meth, and 19% among individuals who reported using meth at their most recent visit.

**Table 1:** Study participants by meth use status and ancestry

	*Never Used Meth* (n, %)	*Ever Used Meth (n, %)*	***Total***	*p-value*
**Sex**				
Male	3606 (75.9%)	1103 (92.2%)	4709 (79.2%)	** < 0.001**
Female	1144 (24.1%)	93 (7.8%)	1237 (20.8%)	
***Age at Visit (mean* ± *SD)***	*39.7* ± *10.7*	*38.1* ± *9.3*	*39.5* ± *10.3*	** < 0.001**
**Ancestry**				
*AFR*	2845 (59.9%)	237 (19.8%)	3082 (51.8%)	0.15
*AMR*	690 (14.5%)	307 (25.7%)	997 (16.8%)	0.15
*EUR*	1215 (25.6%)	652 (54.5%)	1867 (31.4%)	0.15
**Array**				
Illumina Infinium MEG	1398 (29.4%)	235 (19.6%)	1633 (27.5%)	
Illumina MEGA	1484 (31.2%)	484 (40.4%)	1968 (33.1%)	
*Illumina MEGA-Ex*	1868 (39.3%)	477 (39.9%)	2345 (39.4%)	
**Site**				
*CWRU*	511 (10.8%)	21 (1.8%)	532 (8.9%)	
*FENW*	264 (5.6%)	162 (13.5%)	424 (7.1%)	
*JH*	880 (18.5%)	39 (3.3%)	919 (15.5%)	
*UAB*	1571 (33.1%)	161 (13.5%)	1732 (29.1%)	
*UCSD*	628 (13.2%)	340 (28.4%)	968 (16.3%)	
*UCSF*	82 (1.7%)	179 (15.0%)	261 (4.4%)	
*UNC*	529 (11.1%)	43 (3.6%)	572 (9.6%)	
*UW*	285 (6.0%)	251 (21.0%)	536 (9.0%)	
***Dropout Rate***				
	13.0%	15.0%^a^	15.0%	
**Total**	4750 (79.9%)	1196 (20.1%)	5946 (100%)	

Never Used/Ever Used refer to lifetime methamphetamine use. *SD* Standard Deviation, *AFR* African Ancestry, *AMR* Admixed American Ancestry, *EUR* European Ancestry, *MEG* Multi-Ethnic Global-8 Kit, *MEGA* Multi-ethnic Global Array, *MEGAEx* Multi-ethnic Global Array Expanded Version. Sites: *CWRU* Case Western Reserve University, Cleveland, OH, *FENW* Fenway Health Centers, Boston, MA, *JHU* Johns Hopkins University, Baltimore, MA, *UAB* University of Alabama, Birmingham, AL, *UCSD* University of California San Diego, La Jolla, CA, *UCSF* University of California San Francisco, San Francisco, CA, *UNC* University of North Carolina, Chapel Hill, NC, *UW* University of Washington, Seattle, WA

^a^Dropout rate among individuals who reported ever having used meth was 15%. However, the same rate among individuals who reported using meth at their most recent visit was 19%

**Fig. 1:**
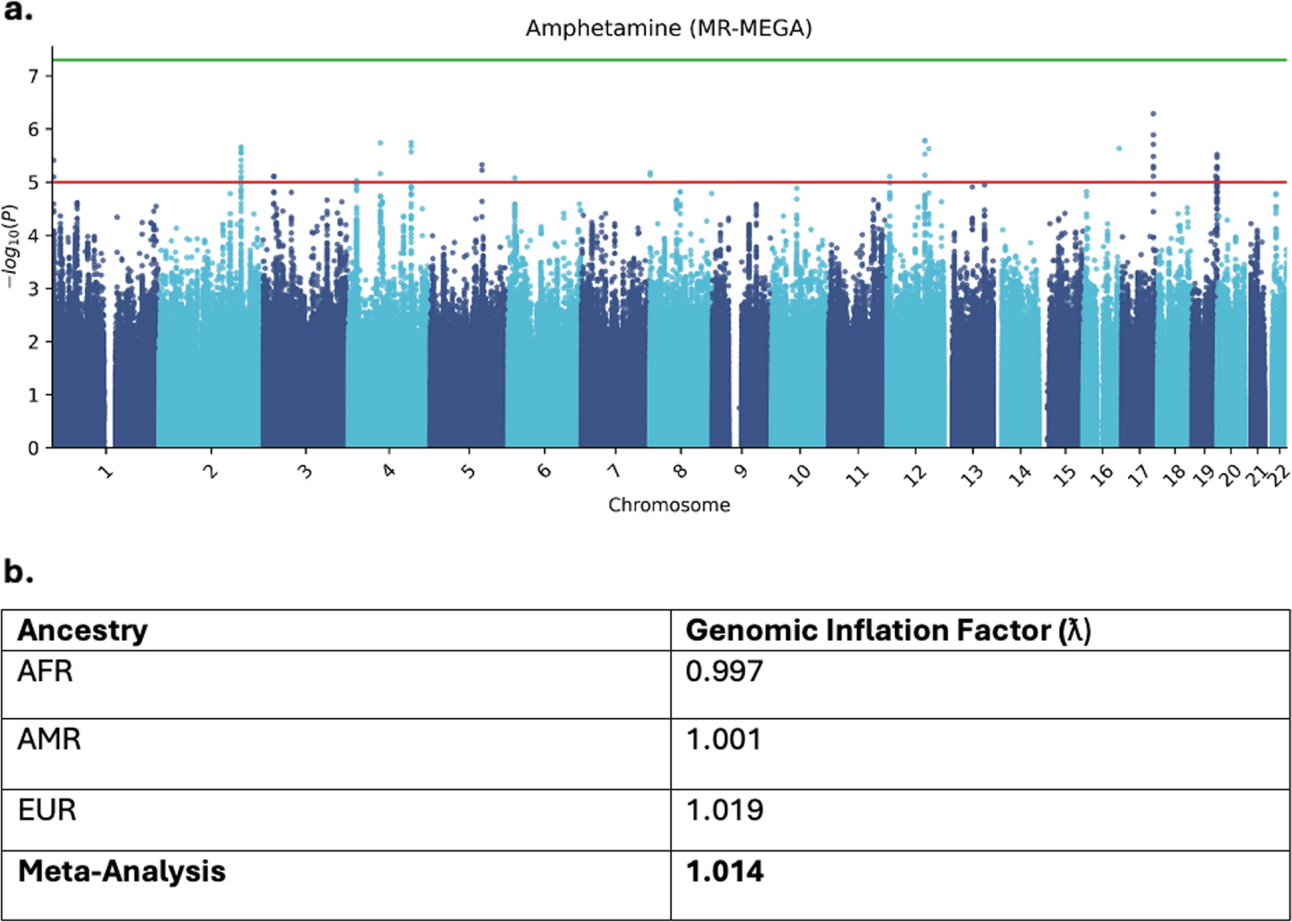
**a** Manhattan plot of SNP signals associated with lifetime methamphetamine use. No SNP reached the genome-wide significance threshold (*p* < 5*10^–8^), represented by the green line. **b** Genomic inflation factors associated with ancestry-specific analyses and meta-analyses for lifetime methamphetamine use

No single nucleotide polymorphism (SNP) reached genome-wide significance (*p* < 5*10^–8^) in the multi-ancestry GWAS (see Fig. 1 and Table 2). The strongest association was observed for the rs55723510 SNP (*p* = 4.95 × 10^–7^). Genomic Inflation Factors (ƛ) were calculated for both single-ancestry studies and the overall meta-analysis (Fig. 1b). All factors were close to unity.

**Table 2:** The 10 strongest SNP associations with lifetime meth use in CNICS

rs ID	Associated Gene/Region	Chr	Position	EA	NEA	EAF	*P*-Value (Total)	OR	95% CI
rs55725310	*SDK2*	17	73437322	A	G	0.24 (Meta) 0.27 (AMR) 0.20 (AFR) 0.30 (EUR)	4.95e-07	0.75	0.73, 0.78
rs35824117	*SDK2*	17	73434952	T	TA	0.25 (Meta) 0.27 (AMR) 0.20 (AFR) 0.30 (EUR)	1.25e-06	0.76	0.74, 0.79
rs1245582024	*-*	12	88340621	A	G	0.24 (Meta) 0.08 (AMR) 0.38 (AFR) 0.09 (EUR)	1.48e-06	0.69	0.63, 0.76
rs10777105	*-*	12	88341917	C	T	0.24 (Meta) 0.08 (AMR) 0.38 (AFR) 0.09 (EUR)	1.52e-06	0.69	0.63, 0.76
rs11654803	*SDK2*	17	73435028	T	C	0.25 (Meta) 0.27 (AMR) 0.26 (AFR) 0.30 (EUR)	1.88e-06	0.76	0.75, 0.78
rs731517	*LINC01095 (intergenic RNA)*	4	146114396	G	A	0.31 (Meta) 0.24 (AMR) 0.36 (AFR) 0.28 (EUR)	1.90e-06	0.77	0.68, 0.87
rs1882396	-	2	190851286	G	T	0.34 (Meta) 0.49 (AMR) 0.29 (AFR) 0.35 (EUR)	1.99e-06	0.78	0.74, 0.83
rs7439202	*ENSG…249,942 (lncRNA)*	4	74589224	T	G	0.16 (Meta) 0.12 (AMR) 0.21 (AFR) 0.10 (EUR)	2.08e-06	0.70	0.61, 0.81
rs6434423	-	2	190853422	C	T	0.34 (Meta) 0.49 (AMR) 0.29 (AFR) 0.35 (EUR)	2.17e-06	0.78	0.74, 0.83
rs146115874	*LINC01095 (intergenic RNA)*	4	146115874	C	T	0.32 (Meta) 0.24 (AMR) 0.37 (AFR) 0.28 (EUR)	2.23e-06	0.77	0.69, 0.87

*EA* Effect Allele, *NEA* Non-Effect Allele, *EAF* Effect Allele Frequency, *OR* Odds Ratio, *CI* Confidence Interval, *Meta* representing results from the meta-analysis, *AMR* Admixed American Ancestry, *AFR* African Ancestry, *EUR* European Ancestry

We compared our results to those previously reported [11–14]. Among the six SNPs previously reported, two (rs4427170, rs102706556) were identified in our GWAS, but neither reached statistical significance (*p* < 0.05) for meth use in our analyses. (see Table 3, below).

**Table 3:** Associations between previously reported SNPs and lifetime meth use in CNICS

Publication	Total Sample Size	Rs	Gene	Chr	Position	EA	NEA	EAF	Observed OR	Reported *p*-value	Observed *p*-value
Ikeda et al. [12]	1,100	rs4427170	*SGCZ*	8	14996272	T	A	0.28 (Meta) 0.41 (EUR) 0.45 (AMR) 0.15 (AFR)	1.05	3.9e-6	0.41 (Meta) 0.44 (EUR) 0.95 (AMR) 0.59 (AFR)
Chang et al. [11]	4,608	rs112706556	*ANKS1B*	12	99494606	A	G	0.19 (Meta) 0.17 (EUR) 0.18 (AMR) 0.21 (AFR)	0.96	1.5e-8	0.52 (Meta) 0.82 (AMR) 0.24 (EUR) 0.59 (AFR)

Reported *p*-value corresponds to the value reported in the original manuscript. Observed *p*-value corresponds to the *p*-value for that SNP found in our analysis. ‘Meta’ corresponds to the EAF and *p*-value found in the overall meta-analysis. *EA* Effect Allele, *NEA* Non-Effect Allele, *OR* Odds Ratio, *EUR* European Ancestry, *AMR* Admixed American Ancestry, *AFR* African Ancestry

## Discussion and conclusion

There are multiple potential explanations for the differences between our results and those of past GWAS of meth use. First, previous GWASs included people with diagnosed meth use disorder rather than people who self-reported lifetime meth use, and a limitation of this study is the lack of detailed information about nuanced meth use beyond never/ever or current use. Previous GWASs differed in their inclusion criteria from ours: for example, *Uhl *et al*.* and *Ikeda *et al*.*required that individuals report meth use over 20 times per year or be an in-/out-patient of a psychiatric hospital. Further, prior meth use GWASs were all performed in East Asian populations in Japan and Taiwan, while our study is set in a multi-ancestry population in the US. That our cohort is comprised of patients from multiple geographically distinct sites may also affect our results, given that different areas of the United States differ in meth availability, use prevalence, and stigmatization [27]. Relatedly, given that meth use continues to be stigmatized, it is possible that patients were not comfortable reporting their use status and that the overall number of cases differs from that reported. A discrepancy between reported and true meth use status may also be represented in dropout rates in our population, particularly given that dropout rates were somewhat higher among individuals who reported using meth at their most recent visit. However, the impact of this discrepancy on our results is mitigated by the fact that our exposure is genetic variation which remains stable throughout the lifetime in contrast to other varying clinical characteristics that may be more impacted by dropout rates. Our population is comprised of PWH, potentially limiting generalizability of our results to people not living with HIV. Our analysis may also be limited by variation in SNP-specific allele frequencies across ancestry groups, though we accounted for this in the meta-analysis [25].

Our top two candidate SNPs (rs55723510, *p* = 4.95e-07; rs35824117, *p* = 1.25e-06) both correspond to the Sidekick 2 (*SDK2)* gene. The Sidekick family of genes (*SDK1* and *SDK2*) belong to the Immunoglobulin superfamily of cell surface proteins, and recent human genetic studies and animal experiments have implicated both in neurodevelopmental and psychiatric disorders [28]. *SDK1* and *SDK2* are 60% identical at the amino acid level, and in vertebrates are expressed by non-overlapping subsets of retinal neurons. While *SDK1* has been associated with addiction in animal models, *SDK2*may be associated with other neurological disorders, including autism spectrum disorders and panic disorders [29–31]. *SDK1*has also been shown to be associated with attention-deficit hyperactivity disorder [32, 33]. The involvement of both*SDK* genes in neurological and psychiatric disorders, including addiction, aligns with their potential association with meth use, and can be further explored through more powerful studies. Further, if *SDK2* variation is also associated with ADHD, any variants highlighted in this study may be associated with ADHD and medical use of meth. However, as we assessed use of specific types of meth including crystal meth, speed, and ‘Tina’, and did not ask about commonly prescribed amphetamines for ADHD (e.g., dextroamphetamine/Dexedrin.

As we continue to generate additional genome-wide genotype data in CNICS, we will increase our statistical power to identify SNPs with low-to-moderate effects on meth use. Nevertheless, this study does not find a single strong genetic contributor to lifetime meth use in the CNICS population. While our finding is not evidence that there is no significant genetic contributor, this finding as well as the discrepancy between our study and previous reports of SNPs associated with meth use disorder warrants larger studies with well-defined phenotypic information.

## Data Availability

The dataset supporting the conclusions of this article is derived from the CFAR Network of Integrated Clinical Systems network administered at the University of Alabama, Birmingham (https://sites.uab.edu/cnics/). Data requests for CNICS data and project propsals may be submitted at https://sites.uab.edu/cnics/submit-a-proposal/.

## Abbreviations

CNICS: Center for AIDS Research Network of Integrated Clinical Systems
GWAS: Genome-wide association study
SNP: Single Nucleotide Polymorphism
PWH: People with HIV
WHO ASSIST: World Health Organization - Alcohol, Smoking, and Substance Involvement Screening Test
GRCh38: Genome Reference Consortium Human Build 38
MEGA(Ex): Illumina Multi-Ethnic Global Array (Expanded)
MEG: Infinium Multi-Ethnic Global-8 Kit
1KGP: 1,000 Genomes Project
AFR: African Ancestry Group
AMR: Admixed American Ancestry Group
EUR: European Ancestry Group
TOPMed: Trans-Omics for Precision Medicine
